# Video Game Addiction and Emotional States: Possible Confusion Between Pleasure and Happiness?

**DOI:** 10.3389/fpsyg.2019.02894

**Published:** 2020-01-27

**Authors:** Lucio Gros, Nicolas Debue, Jonathan Lete, Cécile van de Leemput

**Affiliations:** ^1^Research Center for Work and Consumer Psychology, Université Libre de Bruxelles, Brussels, Belgium; ^2^Department of Psychiatry and Neurosciences, Maastricht University, Maastricht, Netherlands

**Keywords:** video games, addiction, confusion, pleasure and happiness, emotional states

## Abstract

Internet gaming disorder is characterized by a severely reduced control over gaming, resulting in an increasing gaming time and leading to negative consequences in many aspects of the individual life: personal, family, social, occupational and other relevant areas of functioning (World Health Organization). In the last years, the significant boom in using video games has been raising health issues that remain insufficiently understood. The extent of this phenomenon (the estimated prevalence is between 1.7 and 10% of the general population) has led the mentioned Organization to include gaming disorders in the list of mental health conditions (2018). Several studies show converging findings that highlight the common brain activities between substance use disorders and behavioral addictions (i.e., gaming disorders). Addiction specialists observed that addict subjects tend to confuse pleasure with happiness when linking emotional states to their addictive activities. As far as we know, beyond the mentioned observations, distinguishing the perception of these two emotional states in the frame of an addiction has not been yet the object of formal research. This study aims at examining the possible confusion between pleasure and happiness within the addiction sphere. Video game addiction has been chosen to explore the possible occurrence of this perceptional distortion. A mixed design lab-based study was carried out to compare between video games addicts and non-addicts (between-subjects), and video games-related activities and neutral activities (within-subject). Emotional reactions were gauged by self-reported scales and physiological data acquired through a range of biosensors: Relaxation and Hearth Rate. From a therapeutic standpoint, this research intends to explore alternatives to deal with this sort of disorders. More specifically, at the cognitive level, the idea is elaborating guidelines to develop patients’ insights into these emotional states and thus increasing their ability to handle them. Overall, several indices resulting from this study constitute a bundle of arguments that argue in favor of the confusion between pleasure and happiness made by addict users when associating their affective states to video gaming. Furthermore, this approach illustrates how reappraising emotions may contribute to reducing the perceptional distortion of these emotional states.

## Introduction

In the last years, the significant boom in using video games (VG) has been raising health issues that remain insufficiently understood (Khazaal et al., 2016). The World Health Organization [WHO] (2018) has recently included “gaming disorders” in the list of mental health conditions. According to WHO this affliction is a “persistent or recurrent behavior pattern of sufficient severity to result in significant impairment in personal, family, social, educational, occupational or other important areas of functioning.”

The fifth revision of the Diagnostic and Statistical Manual of Mental Disorders (DSM-5) considers the ‘Internet Gaming Disorder’ as a potential new diagnosis that requires further research (Petry et al., 2015). The prevalence of problematic gaming is estimated to range from 1.7% to over 10% among general population (Griffiths et al., 2012).

Compared to the core topics of research in neuroscience such as stress, depression, etc., the chronic use of VG is a rather recent field of investigation. Yet, a growing number of studies have been produced in this field in the last two decades (Andreassen et al., 2016). Indeed, several research projects have been exploring VG addiction from a behavioral, emotional, brain circuits and genetic perspectives (Griffiths et al., 2012; Dong et al., 2017).

There seems to be converging findings that highlight the common brain activities between VG disorders (belonging to the cluster of behavioral addictions) and substance use disorders (SUD). It has been shown that the dorsolateral prefrontal cortex, orbital frontal cortex, para-hippocampal gyrus and thalamus were activated in both disorders (Han et al., 2011). The limbic structures appear to be the key circuits linked with reward and addiction (Cooper et al., 2017). In subjects suffering from these disorders, cues associated with SUD and with behavioral addiction can trigger craving, which is connected with the dopamine reward system (Ko et al., 2009; Han et al., 2011). In addition, it has been observed that the level of dopamine released in the ventral striatum when playing a competition like video game is comparable to that provoked by psycho-stimulant drugs (Koepp et al., 1998; Yau et al., 2012). Few studies have been carried out on the genetic aspects of this topic. Some of them indicate that there would be genetic background similarities between these two disorders. For example, the homozygous short allelic variant of the 5HTTLPR gene (encoding the serotonin transporter) is more prevalent among the excessive Internet user, which is also linked with increased drug consumption (Serretti et al., 2006, as cited in Yau et al., 2012; Lee et al., 2008, as cited in Yau et al., 2012).

As described later, studying the confusion between pleasure and happiness in the frame of addiction requires as clear a demarcation as possible between these two emotional states. Although a consensus among scientists on how to define and distinguish pleasure and happiness remains to be reached (see next section *Pleasure and Happiness*), in this research we have adopted the following distinctive traits to describe and to work with these two emotional states: *pleasure* relates to a transient emotional state resulting from the satisfaction of a desire, a craving, and *happiness* refers to a lasting emotional state of contentment, euthymia (Pollard, 2003; Lustig, 2017).

According to Lustig (2017), addictions together with depression are two rampant afflictions in the last decades and constitute the harmful extremes of pleasure (associated with the dopaminergic system) and happiness (associated with the serotoninergic system) respectively (Üstün et al., 2004; Lepine and Briley, 2011; Szalavitz, 2011; Whiteford et al., 2013; Gowing et al., 2015; Keyes et al., 2015). Based on his long practice on addiction issues, this author argues that confusing pleasure (in the sense of longing, craving, strongly driven by a short term reward) with happiness is linked with SUD and with behavioral addictions (i.e., gambling, eating disorders, excessive use of technology like for example social media and VG, etc.), which could lead to depression (Lawrence et al., 2014). According to the author, confusing pleasure with happiness is related to the growth rate of this disorder insofar as it would encourage seeking immediate gratifications perceived as sources of happiness, which in turn triggers the reward system with the risk to sink into the vicious circle of addiction (Pollard, 2003). Besides, the significant industrial development, through its commercial campaigns, probably tended to lead individuals to equate consumption with happiness (Schmidt, 2016; Lustig, 2017). From a physiological standpoint, the author highlights that an over excited reward system engenders an excess of dopamine (DA) release from the ventral tegmental area, which in return decreases serotonin (5HT) level (associated with depression) (Pollard, 2003; MacNicol, 2016).

Moreover, Lustig underlines that DA and 5HT amino acids (needed for the production of DA and 5HT) share the amino acid transporters, which poses a problem in case of DA amino acid over presence: that is to say, the more amino acids for DA, the less amino acids transporters are available for 5HT amino acids. In short, this DA-5HT unbalance illustrates one of the facets of the DA-5HT interaction in which the low 5HT level, associated with depression, prevents the serotoninergic system to exert its inhibitory role to imped the over drive of the dopaminergic system (Esposito et al., 2008).

Chronic stress and anxiety may further aggravate this problem by increasing the cortisol level and thus creating a loop with dopamine activating the sympathetic nerve system and reinforcing the reward seeking behavior while down-regulating 5HT -1a receptor, which decreases the serotonin signaling and increases the depression likelihood (Lustig, 2017). These findings are in line with studies that associate stress, anxiety and depression with Internet gaming disorders (Wenzel et al., 2009; Griffiths et al., 2012).

Fundamentally, from a phylogenetic standpoint, it is likely that pleasure has contributed more than happiness (Pollard, 2003; Lustig, 2017), which could explain the stronger drive of the short term gratifications over the quest for medium and long term euthymia. In sum, this suggests that identifying the possible confusion between the mentioned emotional states associated with the addictive activities may contribute to deepen the understanding of this sort of disorders and consequently to explore new therapeutic options.

The emotional states (and their consequences) associated with VG as felt and perceived by chronic users led to thorough interrogations of health professionals. Several studies intended to explore this issue by focusing on the individual characteristics of addict players. For instance, the general level of happiness appears to be a firm candidate to predict addiction to VG playing (Hull et al., 2013). In effect, it has been shown that gaming disorders are positively correlated with depression and loneliness and negatively correlated with well-being (Lemmens et al., 2011; Sarda et al., 2016). These two studies relied on a eudaimonic notion of well-being (i.e., life satisfaction, a life well lived). Thus, based on the mentioned definitions of pleasure and happiness, on the semantic net (see Annex) and on the analysis made in the next section (Pleasure and Happiness), in this research well-being is assimilated to happiness due to the considerable common ground shared between these two concepts. In line with these findings, another study highlights the association between high frequency of online gaming with depression and social phobia (Wei et al., 2012). Similar results were found in a study in which, compared with no addict Internet user, Internet addict subjects used to play online games reported significantly more depressive symptoms (Geisel et al., 2015).

From a psychological symptoms standpoint, it has also been observed that when playing VG, addict gamers have a sense of well being or euphoria while playing VG, inability to stop the activity, craving more time at playing VG, feeling empty, depressed, irritable when not playing VG, with all the pernicious consequences these symptoms have on the private, social and professional life (Griffiths, 2008). At glance, the coexistence of well being and craving might come across as paradoxical, although the mentioned work (Lustig, 2017) on this issue provides some elements of answer to this finding.

Using a video game clip as a stimulation trial, it has been studied (Kim et al., 2018) the craving state of chronic users when playing VG through measures resulting from addiction questionnaires and several bio signals such as eye blinking, eye saccadic movements, skin conductance and respiratory rate. The results of this work showed that during the stimulation trial video game there was a decrease of eye blinking rate, eye saccadic movement rate and mean amplitude of the skin conductance response whereas there was a significant increase of the mean respiratory rate.

Another study (Lu et al., 2010; as cited in Kim et al., 2018) focused on a group of individuals with high risks of developing Internet gaming disorders (IGD) and their sympathetic nervous system responses. When using Internet in this experiment, increases were observed in blood volume, body temperature and respiratory rate. Heart rate (HR) has also been used as a reliable indicator of craving in subjects with SUD (Kennedy et al., 2015).

### Pleasure and Happiness

The psychophysiological and brain mechanisms of *pleasure* and *happiness* are quite complex and probably more research is required to better discerning these processes. Some studies have underlined that the hedonic system includes *wanting* and *liking* and each of these two emotional states may operate in a conscious and unconscious mode (Berridge and Kringelbach, 2011). Studies indicate that unconscious *wanting* would function as a conditioned desire involving the nucleus accumbens, ventral tegmental area, hypothalamus and dopamine; on the other hand the unconscious *liking* would relate to a sensory hedonic dimension associated with the nucleus accumbens, ventral pallidum, periaqueductal gray, amygdala, opioids and cannabinoids (Kringelbach and Berridge, 2009; Berridge and Kringelbach, 2013). The same studies show that conscious *wanting* would relate to cognitive incentives, subjective desires and dopamine whereas conscious *liking* would be linked with subjective pleasures, opioids and cannabinoids; both would involve the orbitofrontal cortex, anterior cingulate and insular.

It has been shown that the level of activation of some of the mentioned areas would be altered in subjects with Internet gaming disorders: sensing craving for gaming is associated with an increased activation of the left orbitofrontal cortex (correlated with desire for VG play) and with a decreased activation in the anterior cingulate cortex (probably linked with the reduced capacity to inhibit craving for gaming) (Wang et al., 2017).

There might be a relation between the complexity of these brain circuits linked to these emotional states and the polysemy of these two terms, *happiness and pleasure*, which may contribute to the possible confusion between them. Indeed, the intense interrelation between them finds expression in subtle distinctive features and in some connotations with vague borders, to the extent that these words might be regarded as almost synonyms. The semantic analysis of these two terms produced in this research intends to show their core meanings, their nuances and the possible intersections between them (Procter, 1985). Trying to unravel and to understand these two emotional states is not a recent endeavor. For instance, Greek thinkers approached the notion of *happiness* as a state constituted by two components: *Hedonia* (pleasure) and *Eudaimonia* (a life well lived) (Kringelbach and Berridge, 2009).

Due to its nature, defining and studying *happiness* is a quite uneasy task. Although progress has been made on this rather recent area of study, there is still a lack of consensus when it comes to defining this concept. Some authors distinguish fluctuating happiness (self centered) from durable, authentic happiness (self-transcendent) (Dambrun et al., 2012). Another study uses the value-arousal model on emotions to define it, according to which *happiness* results from a positive valence, high arousal and engaged and satisfied in life (Cipresso et al., 2014). Lustig (2017) emphasizes the time perspective as one of the distinguishing traits between these two emotional states by opposing the short-term logic of *pleasure* to the longer-term characteristics of *happiness*.

These last two studies are quite illustrative of the differences with regard to defining *happiness*, in particular when it comes to including or not *pleasure* in it. Whilst there seems to be a consensus on “life satisfaction,” “connecting with others” and “contentment” as the main traits of *happiness*, it is less clear whether *pleasure* is part of it. Usually, in the literature there are two understandings to articulate these emotional states: either both (*happiness* and *pleasure)* are seen as inseparable concepts or *happiness* is regarded as a state free from distress (‘liking’ without ‘wanting’) (Kringelbach and Berridge, 2010; Berridge and Kringelbach, 2011; Loonen and Ivanova, 2016; Lustig, 2017). Whether or not *pleasure* is included in the definition of *happiness*, to the best of our knowledge there is no study that includes *craving* (intense desire, longing) as a trait of *happiness*.

Thus, based on the mentioned definitions and on the association between craving and arousal (Kennedy et al., 2015), craving for playing VG may subscribe itself within the realm of *pleasure*, but stands outside of the *happiness’* sphere.

Within the frame of this research, *Pleasure* refers to the hedonic reward processes driven by a desire to obtain a gratification that can lead to *craving* in certain circumstances (Berridge and Kringelbach, 2011). *Pleasure* has been associated with the dopaminergic circuit which can, in certain circumstances, function in an addictive mode and can affect also habits, conditioning, motivation and executives functions such as decision making, inhibitory control, etc. (Volkow et al., 2011).

*Happiness* is understood as contentment and euthymic state, in line with a happy emotional state defined by a positive valence and low arousal (Jatupaiboon et al., 2013). Physiologically, this state implies a reposed mind; akin to the relaxation state measured through the brain electrical activity (Teplan and Krakovskà, 2009). In the literature this mood is related to the serotoninergic circuit (Lustig, 2017).

To the best of our knowledge, there is no existing questionnaire focusing on the association between VG and pleasure/happiness. Thus, our study required a preliminary phase to design such self-report tool whose aim is to explore the perceived emotional states (pleasure/happiness) associated with VG play.

As far as we know, distinguishing the perception of these two emotional states in the frame of an addiction has not been yet the object of formal research, hence the reduced literature on this specific issue, in particular the experimental one.

Consequently this research may be seen as a preliminary study, which aims at examining the possible confusion between pleasure and happiness within the addiction sphere. VG addiction has been chosen to explore the possible occurrence of this perceptional distortion. Emotional reactions of VG addicts and VG non-addicts were gauged via self-report scales and physiological data (Heart rate and Relaxation state) acquired by a range of biosensors.

Resulting from the mentioned background, it is hypothesized that addict VG users:

Are likely to confuse the notions of *pleasure* with that of *happiness* when associating their emotional states to VG play.

The results of this study are expected to show that addict VG users associate happiness with VG activities while feeling craving for playing accompanied by an increased HR and a low relaxation level. Given the shortage of previous researches on the specific issue related to the confusion between pleasure and happiness in VG addiction, the outcome of this study is approached in an exploratory manner.

From a therapy standpoint, this project intends to explore alternatives to deal with this kind of scenarios. More specifically, at the cognitive level, the idea is finding means to develop patients’ insights into these emotional states and thus increasing their ability to handle them.

## Materials and Methods

### Preliminary Phase: Design of the “Pleasure and/or Happiness and VG” Questionnaire

#### Participants

In total 105 VG players participated in this survey, out of which 61 filled all the questionnaires required for the design of the “Pleasure and/or Happiness and VG” questionnaire. The mean age of these 61 participants was 24.28 and the standard deviation 5.48. There were 33 males (54.1%) and 28 females (45.9%). The mean of playtime during working days was 4.49 h and the standard deviation 6.82, and during holidays and weekends 4.68 h and the standard deviation 3.13.

#### Procedure

An online survey was run via video game forum and Reddit site (network of communities with common interests). The purpose of this survey was to evaluate the internal coherence of our self-report tool (Pleasure and/or Happiness and VG) relative to two validated questionnaires (on Hedonic tone and Happiness). Thus the survey consisted in filling the three questionnaires. Participants completed anonymously and voluntarily the questionnaires through their online gamers groups.

#### Measures

Two validated and known questionnaires were used to construct the ‘*Pleasure and/or Happiness and VG’* questionnaire through which the emotional states associated with VG activities were evaluated: the Snaith-Hamilton Pleasure Scale (SHAPS) (Snaith et al., 1995), an assessment tool of hedonic tone, and the Oxford Happiness Questionnaire (OHQ) (Hills and Argyle, 2002). The French version of these two questionnaires was used (Loas et al., 1997; Bruchon-Schweitzer and Boujut, 2014).

The abbreviated SHAPS is composed of 14 items to assess the hedonic tone and the absence of it. The answer scale for each item offers four possible options ranging from ‘Definitely agree’ to ‘Strongly disagree.’ The OHQ is extensively used to evaluate the individual level of happiness. For each of its 29 items, the answer scale has 6 options going from ‘Strongly disagree’ to ‘Strongly agree.’

Several items of the SHAPS and the OHQ are quite adapted to the VG paradigm and lend themselves to be contextualized. For example, the first item of the SHAPS questionnaire is formulated as: “I would enjoy my favorite television or radio program.” In this case “television or radio program” is replaced by “video game.” An example of OHQ concerns the item “I am very happy,” which became “I am very happy when playing VG.” So, these kinds of items constitute the questionnaire whose aim is identifying the emotional states that users associate with VG. Initially, eight items were adapted to VG from these two questionnaires: four items from SHAPS and four items from OHQ. The answer scale provides with six possible options ranging from ‘fully disagree’ to ‘fully agree.’

#### Statistical Analysis

In order to ensure the usefulness of the designed self-report tool, an Alpha Cronbach test was run on the results of this survey to measure the internal coherence between the ‘VG and Pleasure/Happiness’ and the two other questionnaires (SHAPS and OHQ). Moreover, it has been examined whether there is a correlation between VG play frequency and the two areas explored in this survey: the general happiness level (OHQ) and the emotional states associated with VG (‘Pleasure and/or Happiness and VG’).

### The Experiment

#### Participants

The study was announced through the Université Libre de Bruxelles (ULB) scientific social media as well as via leaflets available in public cyber games centers in Brussels. Gamers interested to participate in this study had to answer an on-line survey (*N* = 163), in which the following data was gathered: age, play frequency, name of VG played and a validated test to assess the gaming addiction level (Gaming Addiction Scale, Lemmens et al., 2009). The French version of this scale was used (Gaetan et al., 2014). Being used to play to at least one of these five popular VG (Fornite, Overwatch, League of Legends, Counter-Strike or Rocket League) and an age ranging from 18 to 70 years old were the inclusion criteria. Competing against another team and playing in groups are the common characteristics of these VG. The exclusion criteria were having vision impairments and neurological problems.

Two groups of gamers were invited to participate in this study: addict users (AU) and non-addict users (NAU). None of the invitees met the exclusion criteria. The selection and recruitment were based on the score obtained in the test on gaming addiction, resulting in: AU (*N* = 12) and NAU (*N* = 17) (7 females and 22 males, ranging from 19 to 29 years old). They were all French speakers Belgian residents. The mean age was 23 and the standard deviation of 3. The difference between sexes in terms of VG addiction is not statistically significant (3/7 AU females and 9/22 AU males, U 45.5, *p* = 0.130).

#### Procedure

This experiment took place within the frame in the usability laboratory of the Research Centre of Work and Consumer Psychology, Université Libre de Bruxelles (ULB).

Before the experiment all the procedures were explained to participants and their consent was asked on formal basis. They were informed that:

– This experiment aims at better understanding the video game phenomenon (without mentioning the issue relative to the emotional states and VG).
– They have to fill several questionnaires (in French).
– Some non-invasive artifacts are set to gather measurements on physiological signals while they watch video clips.
– The Ethical Committee of ULB approved this study in accordance with the Declaration of Helsinki.

The participants were welcome into the testing room of the laboratory by the examiner. They were seated and given an informed consent form. Once the form was read and signed, the study procedure was explained. Then, the Electroencephalogram (EEG) headset was placed onto the participant’s head and an impedance check was run.

Before the beginning of the experiment, each participant chose his/her favorite VG he/she uses to play among the five initially proposed. During the experiment, the examiner observed the participant through a one-way-glass, avoiding interference.

Finally, participants were thanked for their participation, compensated and given information on obtaining the results of the study. The whole experimental run took around 1 h.

Prior to starting the operational phases of the experiment, all devices are set to initiate the baseline recording of all the physiological signals.

**FIGURE 1:**
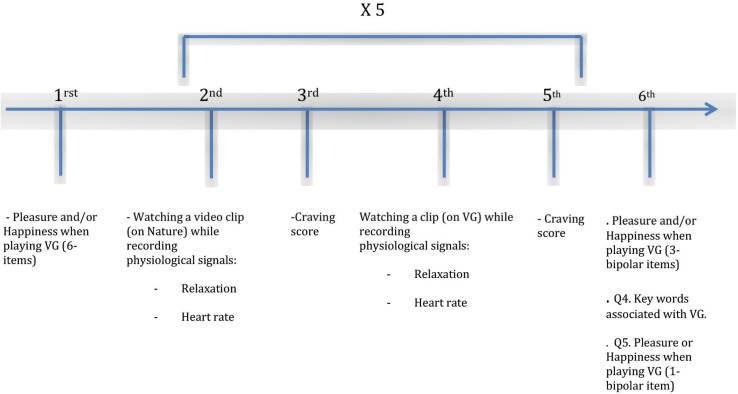
Synthetic view of the experimental phases.

Six phases compose this experiment (Figure 1). In each phase of the experiment the emotional states associated with VG were examined either through self-report questionnaires or via physiological measures. The physiological measures were recorded during the visioning of two sorts of video clips: VG clips whose aim was to induce craving and neutral video clips (documentaries on nature) intending to reduce craving.

The six experimental phases:

(1) “Pleasure and/or Happiness and VG” (six items): Participants were invited to fill the self-report questionnaire designed in the preliminary phase.
(2) Watching a neutral clip during 2 min while recording physiological signals related the mentioned two emotional states. This phase intends to decrease craving in participants.
(3) Craving score: Participants were asked to express their craving state to play their favorite VG via a one item self-report questionnaire.
(4) Watching a VG clip during 2 min while recording the same physiological signals as in phase two related to the mentioned emotional states. The objective of this phase is to increase craving in participants.
(5) Craving score: the same procedure and self-report tool as in phase 3 were applied.
(6) Submission of three self-report questionnaires:
  (6.1) “Pleasure and/or Happiness and VG” (Three bipolar items).
  (6.2) “Key words and VG”: participants were invited to associate a list of words to VG activities.
  (6.3) “Pleasure and VG or Happiness and VG” (one bipolar item): participants were asked to associate one of the two emotional states to VG play.

The cycle from the 2nd phase to the 5th phase was repeated five times for each participant. In each of these five cycles, different episodes of video clips (the chosen VG and the neutral clip) were shown randomly so as to avoid the habituation phenomenon and minimize the influence that the order of the sequence of episodes could have on participants’ responses.

#### Measures

– Experimental groups: AU and NAU

The Gaming Addiction Scale (GAS) (Lemmens et al., 2009; Gaetan et al., 2014) was used to constitute these groups. As a tool to measure game addiction, GAS possesses significant assets. Lemmens et al. (2009) showed the validity of this scale from a cross population point of view and its one-dimensional characteristic resulting from the factorial analysis. In addition, in the same study it has been shown the concurrent validity of GAS insofar as this scale is associated with play frequency as well as with psychological features related with game addiction, namely decreased level of social competence and of well being, and high level of aggression and of loneliness. Moreover, high scores in GAS are also linked with attentional deficiencies in response inhibition when perceiving game cues (van Holst et al., 2012; in Khazaal et al., 2016), which converges with results produced by other researches associating impulsivity and cue reactivity with other addictive behaviors (Billieux et al., 2011; Khazaal et al., 2012; Torres et al., 2013). Relative to other game addiction measurements, GAS has the most complete covering of the Internet gaming disorder criteria of the DSM-5 (Petry et al., 2014). Although it was initially designed for adolescents, there are substantial evidences to state that GAS is applicable for young adults too (Khazaal et al., 2016).

Each of the seven items of this scale starts with the question “How often in the last 6 months…?” to explore the impact of video gaming on different aspects of the subject’s life. The possible answers are: never, rarely, sometimes, often and very often. The first two answers score 0, the last three answers score 1. If the total sum of these scores is 4 or higher, the subject is considered an AU according to this scale.

– The experiment

In the first phase, participants were asked to fill the “*Pleasure* and/or *Happiness* and VG” questionnaire composed by six items: three items that tie Pleasure (P) and VG, three items that tie Happiness (H) and VG (six-items in total).

The answer scale for each item was composed of six options ranging from ‘Fully disagree’ to ‘fully agree.’ Each of these six items is answered separately, thus the overall possible results of this questionnaire can be: (1) P and VG > H and VG or (2) H and VG < P and VG or, (3) P and VG = H and VG.

In the second phase (Neutral video clip), two physiological signals related to Pleasure and Happiness were recorded. Based on the correlates found between HR and craving, this physiological signal is used as an indicator of arousal (Kennedy et al., 2015).

Despite the difficulty in defining and in measuring *happiness*, the brain electrical activity is recorded (Electroencephalogram, EEG) mainly to detect the *relaxation* state. This state appears close to the notion of happiness; in the literature it is accepted that the increase of alpha waves is correlated with mental and physical rest (Teplan and Krakovskà, 2009).

In the third phase, participants were asked to express their *craving* state to play his/her favorite VG. The statement employed in this self-report tool was: “State your present craving for gaming.” Participants have to choose the answer that best fitted their self-assessment among six possible answers offered by the scale ranging from “I do not feel any craving for gaming” to “I feel a very strong craving for gaming.”

In the fourth phase (VG clip), the same physiological signals as in the second phase were measured.

In the fifth phase, the same procedure to assess craving for gaming as in the third phase was employed.

In the sixth phase, three other self-report questionnaires were submitted to participants and used to evaluate the association between the mentioned emotional states and VG:

– “Pleasure and/or Happiness and VG” (three bipolar items). The same six items of the “Pleasure and/or Happiness and VG” questionnaire used in phase 1 were presented in a bipolar structure: three items opposing “Pleasure and VG” vs. “Happiness and VG.” For example, if in the six items questionnaire the items “I would enjoy my favorite VG” (Pleasure/VG) and “I am happy when playing VG” (Happiness/VG) are presented separately, in this questionnaire they are part of the same item: “I would enjoy my favorite VG” vs. “I am happy when playing VG.” By doing so, participants are encouraged to choose which of their emotional states (Pleasure, Happiness) is associated with VG playing. That said, the scale has an uneven number of options (five) between the two extremes, the central option representing the equal association of Pleasure and Happiness with VG play. Thus, the overall possible results are identical as in phase 1.
– “Key words and VG”. Participants were asked to choose three words (out of ten) that they associate most with their VG activities. These 10 key words come from the semantic mapping elaborated in this research of the terms used in the formal statements defining *pleasure* and *happiness* in this study. For example, some words from the *happiness* sphere are *contentment* and *well being*, whereas *desire* and *joy* relate to *pleasure*. Besides, they are in line with both definitions Lustig’s (2017). Only the ten words (French version) were shown to participants. Although the possible results are similar to those of six-item “Pleasure and/or Happiness and VG” questionnaire and three-bipolar item “Pleasure and/or Happiness and VG” questionnaire, this time the same association (emotional states and VG) is tackled via key words directly linked to the two studied emotional states (*Pleasure, Happiness*) but without mentioning them. This self-report format intends to gain accuracy in the identification of gamers’ emotional states associated with VG.
– “Pleasure and VG or Happiness and VG”. The written definitions of both *pleasure* and *happiness*, based on work Lustig’s (2017), were shown to participants. Then they were asked to read carefully these definitions and to take them into account when answering one bi-polar item that opposes “Pleasure and VG” vs. “Happiness and VG.” Unlike in the three-bipolar items questionnaire, the answer scale between these this bipolar item has an even number of options (six). This time is an “either/or” choice they are faced with, therefore the possible results are: P and VG < H and VG or P and VG > H and VG. Basically this questionnaire intends to strengthen consistency in participants’ insights into this issue by inviting them to confront their perception of their emotional states associated with VG play with the mentioned formal definitions, comparable to an emotions reappraisal process (Seay and Kraut, 2007).

In short, four self-report questionnaires (see Annex) aim at exploring this dependent variable (association between these two emotional states and VG play) by looking at the consistency of participants’ answers to the different formats of questions. The questions’ formats are:

– *Pleasure and/or happiness* can be associated with VG (six independent items);
– *Pleasure and/or happiness* can be associated to VG (three bipolar items);
– *Pleasure and/or happiness* can be associated to VG through key words defining the two emotional states (without mentioning the words *pleasure* and *happiness*);
– *Pleasure or happiness* can be associated to VG (written explicit definitions of *pleasure* and *happiness* are given to participants).

This approach aims at exploring the coherence between the self-reported answers and the physiological signals, as a means to objectivize the perceived emotional states associated with VG play by the two mentioned groups of participants (addict gamers and non-addict gamers).

The previously mentioned theoretical framework indicates that the notion of craving relates to an arousal state that could lead to an addictive pattern and consequently stands out of the realm of *happiness.*

#### Expected Results

Based on the analysis made on this issue previously as well as on the hypothesis of this study, the expected results could be synthesized as shown in Table 1.

**TABLE 1:** Summary of the expected results.

**Expected results × group × measurement**	**Happiness and/or Pleasure associated to VG (6-items)**	**Self-report Craving and physiological signals (interaction between the two independent variables on the dependent variable)**	**Happiness and/or Pleasure associated to VG (3 bipolar items)**	**Key words (Happiness and Pleasure) associated to VG**	**Happiness or Pleasure associated with VG (1 bipolar item with definitions)**
Addict Users (AU	Happiness and VG > Pleasure and VG	– VG clip increasing effect on craving – VG clip increasing effect on HR – VG clip decreasing effect on relaxation	Happiness and VG > Pleasure and VG	Happiness and VG > Pleasure and VG	Pleasure and VG > Happiness and VG
Non Addict Users (NAU)	Pleasure and VG > Happiness and VG		Pleasure and VG > Happiness and VG	Pleasure and VG > Happiness and VG	Pleasure and VG > Happiness and VG

– Self-Report Questionnaires

For the self-report questionnaires, it is expected that, compared to NAU, the AU group:

– *In “Pleasure and/or happiness* associated with VG” (six independent items) associates more *happiness* than *pleasure* with VG play.
– Reports more craving for playing after watching VG clip.
– In “*Pleasure and/or happiness* associated to VG” (three bipolar items) associates more *happiness* than *pleasure* with VG play.
– Associates VG play with key words more related to *happiness* category than to those of *pleasure*.
– In “*Pleasure or happiness* associated to VG” associates VG play with *pleasure* (like NAU).

– Physiological Signals

It is expected to observe an interaction between the groups (AU, NAU) and the conditions (VG clip, Neutral clip). Namely, it is assumed that visioning the VG clips has an effect on AU increasing HR while decreasing Relaxation.

#### Statistical Analysis

After verifying the normality of distributions (Kolmogorov–Smirnov), the means comparison between the two groups (NAU, AU) was calculated for self-report questionnaires measuring the association between VG and Pleasure/Happiness (Mann–Whitney *U*) for the six-items “Pleasure and/or Happiness and VG,” the three-bipolar items “Pleasure and/or Happiness and VG” and the one-bipolar item “Pleasure and VG or Happiness and VG.” The Chi square was used for “Key words and VG.” In order to determine whether there are differences between independent groups over time and to identify possible interactions between the two independent variables on the dependent variables, a two-way mixed ANOVA (within and between subjects) was used for the craving scores and the physiological signals recorded (Table 2).

**TABLE 2:** Synthetic view of independent and dependent variables.

	**Phase 1: Self-report**	**Phases 2 to 5: Visioning video clips (Neutral and VG) x Craving score and physiological signals**	**Phase 6: Self-report**
– Addict Users (AU) – Non-Addict Users (NAU)	– Happiness and/or Pleasure associated to VG (6-items)	– Heart Rate and Relaxation – Craving score	– Happiness and/or Pleasure associated to VG (3-bipolar items) – Key words associated to VG – Happiness or Pleasure associated to VG (1 bipolar item)

##### Material

The experiment was run on a desktop computer with an Intel Core i7 quad processor and 8 GB RAM, running Windows 10. Stimuli were displayed on a 22-inch monitor and resolution was set to 1680 × 1050. Participants used standard mouse and keyboard as input devices. EEG measurement includes detecting the fluctuation of voltage potential generated by large group of neurons in the brain. The EEG signal was obtained through the use of EPOC headset. This device allows to remotely getting data of brain activity using a wireless set of fourteen electrodes (AF3, AF4, F3, F4, F7, F8, FC5, FC6, T7, T8, P7, P8, O1, O2) sampled at 128 hertz.

The relaxation state was measured by one of the composite metrics of the Emotiv software. HR was measured by using Schimer 3 (Photoplethysmography). The I. Motions software version 7.1 (Imotions Inc. 2018) was used to recording the mentioned data and presenting stimuli to participants. The statistical analysis was conducted with IBM SPSS statistics v.25.

## Results

### Design of the “Pleasure and/or Happiness and VG” Questionnaire

The Cronbach’s alpha (0.859) showed a high internal coherence between the SHAPS and three items (out of four) of the “Pleasure and VG” within the “Pleasure and/or Happiness and VG” questionnaire. The fourth item has been disregarded; its presence would have dropped the Cronbach’s alpha to 0.685. The internal coherence obtained between the OHQ and the “Happiness and VG” items within the “Pleasure and/or Happiness and VG” questionnaire was quite high for the four items concerned (alpha 0.901). However, the internal coherence between these four items was too weak due to one item (alpha 0.407). The exclusion of this item raised the alpha significantly (0.836). Consequently, only the consistent items have been kept (six out of the initial eight items: three on “Pleasure and VG,” and three on “Happiness and VG,” see Annex).

Moreover, it has been examined whether there is an association between VG play frequency and the two areas explored in this survey: the general happiness level (OHQ) and the emotional states associated with VG via the “Pleasure and/or Happiness and VG” questionnaire. The constitution of the group of frequent gamers and that of non-frequent gamers was determined by calculated median (18 h per week). In line with several studies linking problematic gaming and well-being and life satisfaction, a moderate negative correlation (*R* = −0.249; *p* = 0.056) was found between VG high play frequency and the OHQ scores (Griffiths, 2008; Lemmens et al., 2011). In addition, there is a marginal significant difference [*T*(58) = 1.923; *p* = 0.059] between frequent VG users and non-frequent VG users relative to the OHQ scores.

### The Experiment

#### The “Pleasure and/or Happiness and VG” Six-Items Questionnaire

The Kolmogorov–Smirnov outcome indicates the need for using a non-parametric test to compare the two groups. The Mann–Whitney test shows that there was no significant difference observed between the AU and NAU relative to association between VG play and pleasure (item 1. *U* = 78, *p* = 0.30; item 3. *U* = 75, *p* = 0.24 and item 5 *U* = 86, *p* = 0.49) (Table 3).

**TABLE 3:** Descriptive statistics of “Pleasure and/or Happiness associated with VG” (6-items): [Pleasure (P), Happiness (H) associated with VG].

	**Addiction Bool**	***N***	**Mean Rank**	**Sum of Ranks**
Item 1 P/VG	NAU	17	13.69	231.00
	AU	12	17.00	294.00
Item 2 H/VG	NAU	17	11.35	193.00
	AU	12	20.17	242.00
Item 3 P/VG	NAU	17	13.41	228.00
	AU	12	17.25	207.00
Item 4 H/VG	NAU	17	12.18	207.00
	AU	12	19.00	228.00
Item 5 P/VG	NAU	17	14.06	239.00
	AU	12	16.33	196.00
Item 6 H/VG	NAU	17	11.03	187.50
	AU	12	20.63	247.50
Mean P/VG	NAU	17	12.88	219.00
	AU	12	18.00	216.00
Mean H/VG	NAU	17	10.59	180.00
	AU	12	21.25	255.00

In contrast, there is a significant statistical difference in the three items where AU associate VG play with *happiness* (item 2. *U* = 40, *p* = 0.005; item 4. *U* = 54, *p* = 0.034 and item 6. *U* = 34, *p* = 0.002) more than NAU.

#### Craving Scores

**FIGURE 2:**
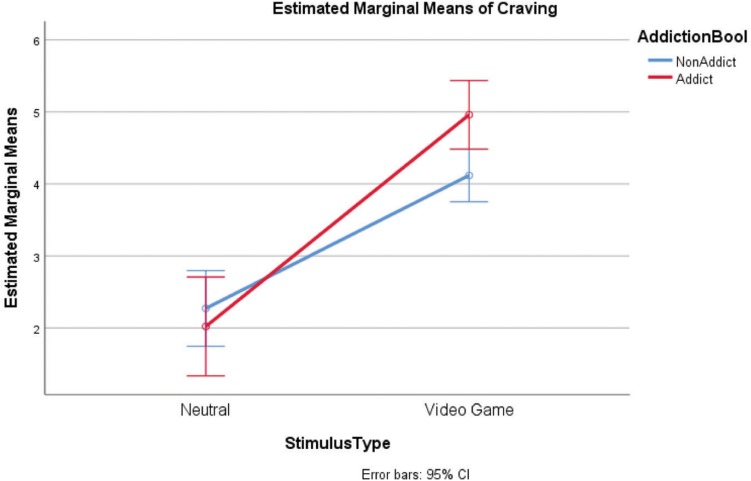
Self-report craving (groups: AU, NAU; conditions: Neutral clips, VG clips).

Results in craving (Table 4 and Figure 2) show a statistically significant interaction *F*(1,25) = 4.78 (*p* = 0.038). Indeed, relative to the neutral clip, the VG clip condition has significantly amplified the reported craving difference between the two groups (AU craving score > NAU craving scores).

**TABLE 4:** Descriptive statistics for self-report Craving.

**Craving/Clips**	**Addiction Bool**	**Mean Craving**	**Standard Deviation**	**Skewness**	**Kurtosis**	***N***
Neutral clips	NAU	2.27	1.09	0.222	–0.954	17
	AU	2.02	0.98	1.617	2.567	10
	Total	2.17	1.03	1.062	1.248	27
VG clips	NAU	4.11	0.82	–0.169	0.135	17
	AU	4.96	0.52	–2.523	7.414	10
	Total	4.42	0.82	–1.271	2.528	27

#### Physiological Signals Measurements

**FIGURE 3:**
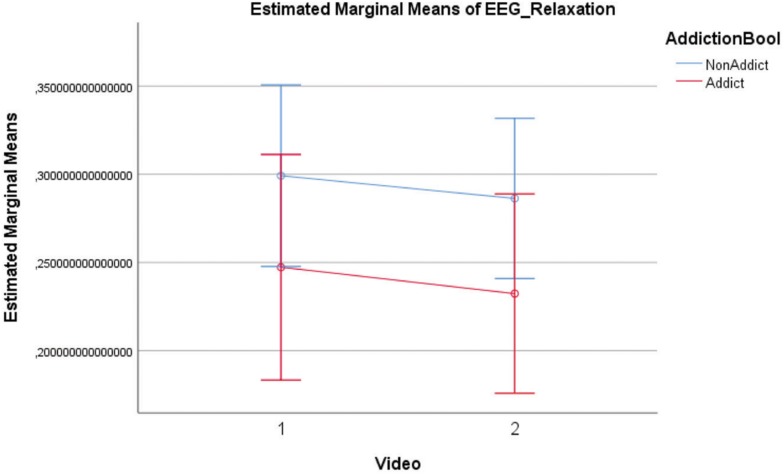
Relaxation [groups: AU, NAU; Conditions: (1) Neutral clips, (2) VG clips].

The AU’s relaxation is significantly lower [*F*(1,24) = 8.616; *p* = 0.007] than NAU’s in both conditions (Between-Subjects Effects). The relaxation level decreases in both groups during the VG clip. On the other hand, conditions do not influence the relaxation difference between the two groups [*F*(1,24) = 0.001; *p* = 0.98] (Table 5 and Figure 3). Furthermore, there is a significant statistical gender difference in both conditions (Neutral clip: Male 17.36, Female 7.57. *U* = 25, *p* = 0.008 – VG clip: Male 17.09, Female 8.43. *U* = 31, *p* = 0.019).

**TABLE 5:** Descriptive statistics: Relaxation index (EEG EPOC, Emotiv software).

**Relaxation/Clips**	**Addiction Bool**	**Mean Relaxation**	**Std. Deviation**	**Skewness**	**Kurtosis**	***N***
Neutral clips	NAU	0.33	0.07	–0.873	1.095	15
	AU	0.24	0.09	1.256	3.303	11
	Total	0.29	0.08	0.292	0.460	26
VG clips	NAU	0.31	0.05	–1.380	1.390	15
	AU	0.23	0.07	1.633	4.688	11
	Total	0.28	0.07	0.292	2.630	26

**FIGURE 4:**
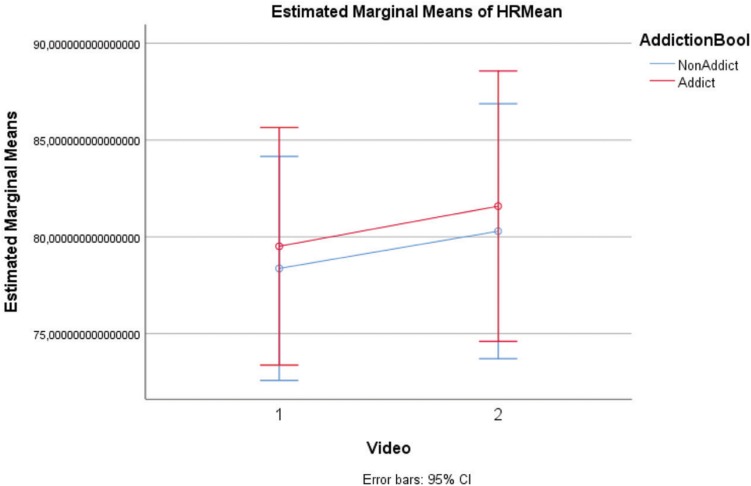
Heart Rate [groups: AU, NAU; Conditions: Neutral clips (1), VG clips (2)].

Concerning the other physiological variable (HR) (Table 6 and Figure 4), there is an effect of VG clips on both groups [*F*(1,15) = 20.802; *p* < 0.001]. Nevertheless, there was no statistically significant interaction [*F*(1,15) = 0.028; *p* = 0.86], nor an effect of addiction on VG clip condition [*F*(1,15) = 0.083; *p* = 0.777]. It is important noting that due to corrupted data the number of valid subjects taken into account was 17 (8 AU and 9 NAU).

**TABLE 6:** Descriptive statistics: Heart Rate (HR).

**Heart Rate/Clips**	**Addiction Bool**	**Mean HR**	**Standard Deviation**	**Skewness**	**Kurtosis**	***N***
Neutral clips	NAU	78.36	7.94	0.054	–0.292	9
	AU	79.51	8.36	2.130	5.013	8
	Total	78.90	7.90	0.972	1.530	17
VG clips	NAU	80.29	9.20	–0.502	0.219	9
	AU	81.58	9.34	2.037	4.661	8
	Total	80.89	8.99	0.614	1.535	17

#### The “Pleasure and/or Happiness and VG” Three-Bipolar Items Questionnaire

The descriptive statistics of this three-bipolar items questionnaire (Table 7), indicate that the AU group linked VG activities more with *happiness* than the NAU group. The Mann–Whitney test shows a significant difference between these two associations (*U* = 47; *p* = 0.013).

**TABLE 7:** Descriptive Statistics: Pleasure/VG vs. Happiness/VG (3 bipolar items).

	**Addiction Bool**	***N***	**Mean Rank**	**Sum of Ranks**
Mean	NAU	17	11.76	200.00
	AU	12	19.58	235.00
Bipolar item 1	NAU	17	13.29	226.00
	AU	12	17.42	209.00
Bipolar item 2	NAU	17	13.91	236.50
	AU	12	16.54	198.50
Bipolar item 3	NAU	17	13.3	221.50
	AU	12	17.79	213.50

#### Key Words and VG

Results state the absence of significant difference between AU and NAU in associating the key words from the Pleasure cluster with VG play, and words from the Happiness cluster with VG (Chi square, *p* = 0.942) (Table 8). When taking words separately, the biggest gap between the two groups relates to the word *well-being* (belonging to the *happiness* cluster) associated to VG play (AU: 25%, NAU: 0%).

**TABLE 8:** Descriptive statistics: number of words per category (Pleasure, Happiness) associated to VG play chosen by NAU and AU.

**% of words × category**	**Addiction Bool**	**Words × subject**	**Pleasure governed by desire**	**Pleasure governing desire**	**Happiness**	**Total**
%	NAU (N17)	17 × 3 words = 51	19.60	47.05	33.33	100
%	AU (12)	12 × 3 words = 36	22.22	47.22	30.55	100

#### “Pleasure and VG or Happiness and VG” (One Bipolar Item Questionnaire With Written Definitions)

The outcome of this questionnaire indicates that there is no significant difference between AU and NAU (*U* = 102, *p* = 1). Both groups have clearly associated VG play with pleasure (Table 9).

**TABLE 9:** Descriptive statistics: Happiness/VG or Pleasure/VG (1 bipolar item, with Definitions of Pleasure and Happiness shown to subjects).

**H/VG or P/VG**	**Addiction Bool**	**Mean**	**Standard Deviation**	***N***
	NAU	4.82	0.636	17
	AU	4.58	1.379	12

The following scheme summarizes the outcomes of the self-report tools used to evaluate the association between the emotional states (Pleasure and Happiness) with VG play (Table 10).

**TABLE 10:** Synthetic view of self-report results (Emotional states associated with VG play).

	**Pleasure and/or Happiness linked with VG (6-items)**	**Pleasure/VG vs. Happiness/VG (3 bipolar items)**	**Key words associated with VG**	**Pleasure/VG or Happiness/VG (1 bipolar item)**
Results	AU associated more Happiness to VG than NAU (Significant difference)	AU associated more Happiness to VG than NAU (Significant difference)	Both groups associated Pleasure and Happiness to VG (No significant difference)	Both groups associated Pleasure to VG (No significant difference)

The following table indicates the mean, standard deviation and Skewness and Kurtosis values of the self-report craving, the HR and the relaxation level for both groups in the two conditions (Table 11).

**TABLE 11:** Descriptive statistics for self-report Craving, Relaxation, Heart Rate.

	**Addiction Bool**	**Mean Craving**	**Standard Deviation**	**Skewness**	**Kurtosis**	***N***
**Craving/Clips**						
Neutral clips	NAU	2.27	1.09	0.222	–0.954	17
	AU	2.02	0.98	1.617	2.567	10
	Total	2.17	1.03	1.062	1.248	27
VG clips	NAU	4.11	0.82	–0.169	0.135	17
	AU	4.96	0.52	–2.523	7.414	10
	Total	4.42	0.82	–1.271	2.528	27
**Relaxation/Clips**						
Neutral clips	NAU	0.33	0.07	–0.873	1.095	15
	AU	0.24	0.09	1.256	3.303	11
	Total	0.29	0.08	0.292	0.460	26
VG clips	NAU	0.31	0.05	–1.380	1.390	15
	AU	0.23	0.07	1.633	4.688	11
	Total	0.28	0.07	0.292	2.630	26
**Heart Rate/Clips**						
Neutral clips	NAU	78.36	7.94	0.054	–0.292	9
	AU	79.51	8.36	2.130	5.013	8
	Total	78.90	7.90	0.972	1.530	17
VG clips	NAU	80.29	9.20	–0.502	0.219	9
	AU	81.58	9.34	2.037	4.661	8
	Total	80.89	8.99	0.614	1.535	17

## Discussion

Overall, the results of this study show that AU associate happiness to VG while reporting craving for VG play and having a low relaxation level. These outcomes observed in this experiment constitute a bundle of arguments that argue in favor of the hypothesis of this study (Lustig, 2017). Indeed, in AU, the high self-report craving score and low Relaxation level during VG clips visioning do contrast with their association of VG more with *happiness* than with *pleasure* in the mentioned “Pleasure and/or Happiness and VG” questionnaires (six-items and three-bipolar-items) relative to NAU. Consistent with previous findings in this area, these four measurements highlight the coexistence of the perception of *happiness* linked with VG play combined with elements related to *pleasure* such as craving (*strong desire, wanting*) (Pollard, 2003; Griffiths, 2008; Waterman et al., 2008). Since craving and low Relaxation are rather incompatible with the mentioned notion of *happiness* (Pollard, 2003; Waterman et al., 2008; Lustig, 2017), these indices may raise the question as to how accurate are AU’s insights into their emotional states associated to VG play and may support the idea that AU’s perception of their emotional states is somewhat distorted. In the literature, VG addiction would be linked with impairment in the self-regulation process, this finding may be linked with the difficulties AU have to observe and evaluate their own behavior (Seay and Kraut, 2007). Besides, the mentioned results suggest that VG clip effect on self-report craving would depend on the addiction level.

Considering that sensing *happiness* and craving are probably experienced as positive emotions by AU, and that usually negative and positive emotional events are reported to last longer and shorter respectively (Gil and Droit-Volet, 2012; Tian et al., 2018), the arousal triggered by motivating stimuli, may modify the time perception and could mediate the effect of emotions on behavior (Gil and Droit-Volet, 2012). In other words, the level of excitement produced by VG play could make AU underestimate the time spent at this activity, which may be perceived as an alleviating evasion free from stressors and possibly assimilated with the notion of *happiness*. This hypothetic mechanism would match one of the possible motives for online gaming (Demetrovics et al., 2011). In this sort of precognitive process, several studies mentioned the involvement of the amygdala in interaction with the thalamus together with the dopaminergic system and a poor inhibitory control (Gil and Droit-Volet, 2012; Petry et al., 2015).

It is noteworthy underlining that the bipolar structure of the three-items questionnaire increases the relevance of this outcome. In effect, although participants were incited to choose between the two emotional states opposing each other (VG and pleasure vs. VG and happiness), like in the six-items questionnaire, AU again did choose *happiness* as the main emotional state linked with VG play. This outcome would further state the difference between these two groups when it comes to associating the two emotional states to VG play. Besides, this would reveal to an important extent that the possibility whereby *pleasure* and *happiness* were regarded as synonyms could be overcome. In other words, this outcome shows that the similarity of meanings of these two concepts did not prevent these groups to make a clear choice. Finally, the similar scores obtained in the two questionnaires (six-items and three-bipolar items “Pleasure and/or Happiness and VG”), in spite of the different disposition of the same items in these two instances, strengthen the value of the designed scale (“Pleasure and/or Happiness and VG play”).

The absence of interaction between the two independent variables on HR may be explained by the fact that a higher arousal would take place in AU when playing VG rather than when watching at VG clips. Moreover, the reduced number of valid subjects when measuring this physiological parameter (due to technical recording problems) could have contributed to this outcome too. The fact that the independent variables did not produce the expected different HR effects on AU and NAU could also be linked with one of the limitations of this study: the difficulty in integrating in this research the interaction between HR and depression (as mentioned, VG addiction is positively correlated with depression) (Griffiths et al., 2012) that may lead to HR index modifications (Cipresso et al., 2014). In sum, this issue illustrates that the difficulty to circumscribe the notion of happiness is also reflected in the complexity to establish physiological correlates so as to objectify this emotional state (Cipresso et al., 2014).

Associating the clusters of key words with VG did not produce the expected results. Since AU linked VG with both *pleasure* and *happiness*, may be these words played a clarification role and facilitated Au’s insights into their emotional states when playing VG. It could also suggest the inadequacy of this self-report tool. However, it is probably worthwhile mentioning an index related our hypothesis: when taking words separately, the word “well-being” associated with VG play was chosen by 25% of AU and by 0% of NAU.

The outcome of the binary question in the “Pleasure and VG or Happiness and VG” one-item questionnaire with the definitions of pleasure and happiness (Pollard, 2003; Deci and Ryan, 2008; Waterman et al., 2008; Kashdan et al., 2008; Lustig, 2017) shows that AU ceased associating *happiness* to VG play and instead, like NAU, clearly linked *pleasure* to their cyber activity. Caution is required in the analysis of these results because the validity of this questionnaire remains to be demonstrated. Having instructed participants to answer the bipolar question by taking into account the written definitions of the two measured emotional states, did modify the result of AU group relative to both questionnaires (“Pleasure and/or Happiness and VG” six-items and three bipolar items). Within the framework of this careful approach, it could be hypothesized that explicit definitions of the two emotional states induced AU to adopting an introspection mode through a more pronounced involvement of cortical brain structures, akin to a therapeutic process in which the appropriate verbalization of *pleasure* and *happiness* facilitates the clarification of one own feeling as a prerequisite to elaborate more adaptive behavior in spite of the constraining psychological characteristics usually associated with VG addicts (Kim et al., 2007; Kashdan et al., 2008; Wenzel et al., 2009).

This may be regarded as an example of emotions reappraisal which would increase accuracy of insights into one-self, reduce distorted perception of emotions and assess the adequacy of the behavioral response to a given stimulus (Compare et al., 2014). In other words, it could be posited that the mentioned explicit definitions have somewhat constrained AU to use a cognitive approach to examine their emotional states related to VG play rather than merely relying on the sensory information as it tends to occur when sensing craving for video gaming (Wang et al., 2017).

Moreover, the result of this one-item binary questionnaire would further support the hypothesis. In effect, the studied interrelation between hedonia and eudaimonia suggests that a highly rated hedonic activity (VG play in this case) is usually related with low rating in eudaimonia (Waterman et al., 2008). This interpretation would fit with the resounding association between depression and gaming disorders (Lemmens et al., 2011; Hull et al., 2013; Sarda et al., 2016; Bonnaire and Baptista, 2019) together with the confusion between pleasure and happiness occurring in addictive activities (AU associated VG with happiness in the first two self-report questionnaires and ended linking pleasure with VG in the last one-item questionnaire) (Pollard, 2003; Lustig, 2017).

**FIGURE 5:**
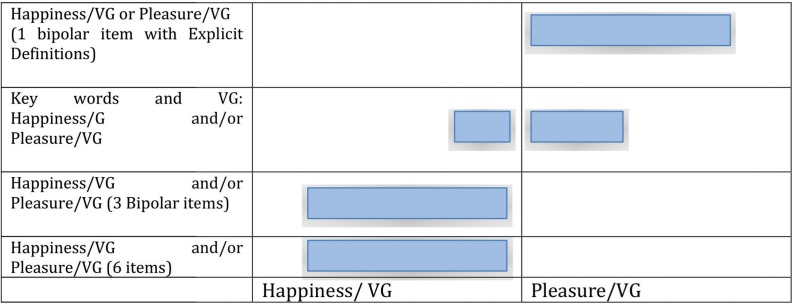
Shift of AU perception of their emotional states associated with VG according to the self-report tools.

Overall, the more explicit the definition of pleasure and happiness and the narrower the choice offered by the self-report questionnaires, the less confusion of emotional states associated with VG occurred in AU group members whereas NAU invariably associated pleasure to VG as illustrated in Figure 5.

Based on these results, it could be postulated that the tendency of AU to perceive *happiness* when feeling craving and *pleasure* linked to VG play, might be moderated by a clarifying cognitive process on the meaning of these studied emotional sates, which would interfere with the behavioral habits linked to the urge of gaming (Ko et al., 2009).

The findings resulting from “Pleasure and/or Happiness and VG” six-items questionnaire could be regarded as an illustration of the confusion that AU might have when linking the studied emotional states with VG play. Unlike NAU, the significantly higher association between VG play and *happiness* expressed by AU matches the perceived level of well being reported by individuals with Internet gaming disorders (Griffiths, 2008). On the other hand, apart from *well-being*, the same author cites *euphoria* as the other main emotional state that addict gamers may report when playing VG. Whilst happiness and well-being rely on each other to define themselves, euphoria would convey the notion of intense excitement, which would rather stand in the pleasure sphere. Moreover, in medical terms, euphoria refers to a feeling of great elation, not necessarily founded (especially when resulting from substances consumption). Since AU also associated VG with pleasure although they did it to a lesser extent than with happiness, it could hypothesized that the feeling of intense excitement derives, at least partially, from satisfying the craving for VG play, which in turn could engender relieve experienced as a sense of well-being (Loonen and Ivanova, 2016).

The impact of VG clips on AU craving and relaxation scores underlines relevant aspects of this study, which support the hypothesis of this research. First of all, it highlights the incongruent perception of AU’s emotional states whereby both craving and *happiness* coexist as emotional states associated with VG play. Thus, this finding constitutes a relevant component of the confusion that consists in placing a short-term pleasure (VG play) within the sphere of happiness. Besides, the low relaxation state of AU would correspond with their self-reported craving and, therefore, further highlights the contrast between the perceived *happiness* associated with VG play and the indicators measured during the VG clip visioning (high craving level and low relaxation state level). Finally, it is noteworthy mentioning that relaxation was the only measure in this study where gender differences were observed. The lower relaxation level in female gamers in both conditions might be related to the gender expectation about playing VG in society at large and in the gamers’ community in particular (Shen et al., 2016). Indeed, since female gamers are a minority in these sorts of VG (Shen et al., 2016) (in line with our sample: 7 females, 22 males), it could be posited that they feel under scrutiny in an activity regarded as male oriented.

### Putative Reasons of Distorted Perceptions of Emotional States Associated With VG Addiction

The social dimension of popular VG has been identified as one of the factors that may explain the addiction pattern (Hull et al., 2013). In this kind of competitive games, improving the required abilities and obtaining better results would be part of the key motives for VG play (Demetrovics et al., 2011), that usually generates the appreciation and the acceptance of the other group players. Getting this sort of feedback from others can be motivating indeed, especially when taking into account the correlation between IGD and social isolation, low self-esteem, traumatic experiences, depression and low life satisfaction (Petry et al., 2015; Schimmenti et al., 2017; Bonnaire and Baptista, 2019). In turn, these psychosocial characteristics are probably related also with the high impulsivity level in VG addicts (Billieux et al., 2011), which has been found to be associated with difficulties in interpersonal relationships (Ryu et al., 2018). Thus, it would seem that VG activities are, at least partially, sating the mentioned social and psychological deficiencies. This suggests that AU’s emotional states related to VG play may be quite contrasting, in which components of *happiness* (i.e., interacting with others, fellowship and belonging to a group) are intertwined with those of short-term pleasure (i.e., craving for getting quick results, praise from others, etc.) (Loonen and Ivanova, 2016). Now, craving for undertaking these cyber activities to respond to the mentioned social isolation issues places this emotional state much closer to the ‘pleasure governed by desire’ than to ‘atmosphere of good fellowship’ (Happiness) (Lawrence et al., 2014; Lustig, 2017).

The flow, defined as the emotional state embracing perception distortion and enjoyment produced by VG activities, is another element that can create confusion in gamers’ insights into their emotional states (Chou and Ting, 2003; Hull et al., 2013). As described in the mentioned study, experiencing flow implies not only losing the notion of time but also merging oneself with the VG actions. In these conditions, the gamer’s senses and attention are in the *here and now*, with little or no awareness about sources of stress relative to past, present or future events. In this line, the motivation to experience immersion has been associated with problematic gaming (Billieux et al., 2011). Considering the fact that loneliness and depression have been identified as predictors of VG addiction and of Internet Gaming Disorders (Hull et al., 2013; Sarda et al., 2016), it is understandable why in gamers’ mind experiencing flow could equate this feeling with a relieving emotional state (Loonen and Ivanova, 2016). This sense of alleviation could match the notion of *happiness* as free from distress (Kringelbach and Berridge, 2010; Loonen and Ivanova, 2016) if it resulted from the quality of real life being lived. Instead, in AU, this relieving and enjoyable emotional state would be engendered by a virtual activity (VG), possibly used as a means to escape from stress and to forget tensions (Demetrovics et al., 2011; Bonnaire and Baptista, 2019). In the literature, the escaping strategy is a way to find relieve from stressors through the engagement in a pleasant activity, which may end up representing a space of *happiness* (Seay and Kraut, 2007).

In sum, the incongruence lies in the coexistence of regarding VG as a space of *happiness* while using VG to get quick pleasures and relief. Individuals suffering from this disorder tend to pursuit short-term pleasures rather than long-term gains (Dong and Potenza, 2015). Being driven by short-term gratifications rather belongs to the reward-seeking realm (Waterman et al., 2008; Lustig, 2017). Thus, this pleasant emotional state could be associated with the arousal linked to a reward seeking behavior through which quick and positive results are obtained, which in turn reinforce the mentioned behavior. Probably, this intense arousal situates itself within the sphere of *pleasure* as a dysfunction in the rewarding system (Pollard, 2003; Berridge and Kringelbach, 2013; Lustig, 2017) and not in that of *happiness* in spite of the relieving benefits it provides.

Another possible reading on why the emotional states generated by these cyber activities are linked with happiness may be related to the way in interpreting the experienced sensations. This representation is probably shaped by the individual background, experiences, culture, etc. From a brain mechanism stand point, conscious liking does not limit it self to a sensory outcome, it is also translated into a subjective liking through the recruitment of cognitive processes (Berridge and Kringelbach, 2013). Indeed, these authors state that conscious pleasure rating is sometimes detached from affective reactions as people can elaborate reasons to themselves for how they should feel. Therefore, associating VG with *happiness* may be the result of a rationalization process to reduce the cognitive dissonance. In other words, the unwished consequences of the VG addiction pattern (increased stress, problems at working, studying, socializing, etc.) (Griffiths et al., 2012) probably produce an increasing amount of pressure (due to the difficulty to reduce gaming time, guilt, etc.) that can become overwhelming if it lasts too long. Consequently, if the affected individuals are unable to master the yearning for VG, perceiving VG activities as a source of well being may reduce the mentioned pressures insofar as the notion of *happiness* usually suggests a socially acceptable mood, a legitimate aim and a safe emotional state. In this perspective, equating happiness with satisfying craving and with short-term pleasure might contribute to feed the addictive pattern (Lustig, 2017).

In a broader perspective, the rationalization process described in the previous paragraph may be also related with coping strategies to deal with adversity. For instance, it has been observed that problematic gamers may use VG play as a means to cope with stressors and to enhance mood (Demetrovics et al., 2011). An association has been found between stressful life events and addiction to Internet activities (Schimmenti et al., 2017), with the mediating role of psychological needs satisfaction and the moderating role of coping styles (Dongping et al., 2016). Several theories and studies support this approach that strives for a more holistic understanding of this issue. The self-determination theory postulates that humans share three universal psychological needs (Deci and Ryan, 2000; in Dongping et al., 2016): autonomy (i.e., feeling of being self-determining in one’s behavior), relatedness (i.e., the feeling of connectedness to others) and competence (i.e., the feeling of dealing with issues in a competent manner). Besides, individuals can adopt different strategies to cope with adversity (Lazarus and Folkman, 1984; in Dongping et al., 2016). According to Zheng et al. (2012; in Dongping et al., 2016), the positive coping approach is the set of strategies aiming at problem solving, support seeking and cognitive restructuring to address the stressors. On the other hand, according to the same authors, the negative coping consists in strategies such as blaming, social withdrawing, denial and disengagement so as to avoid the stressful situations. Now, a parallel can be established between these two coping styles and the brain activities involved in the goal-directed learning and the habit learning.

The goal-directed learning would correspond to the positive coping style insofar as it focuses on the relationship between an action and the motivational value of the outcome, and is associated with the activation of the prefrontal cortex, the dorsomedial striatum and the dorsomedial thalamus (Ballaine and Dickinson, 1998; in Schwabe et al., 2012). On the other hand, habit learning, would be linked with the avoidant coping style. This learning process encodes the relationship between a response and preceding stimuli without taking into account the outcome caused by the response and is related to the activation of the dorsolateral striatum (Yin et al., 2004; Tricomi et al., 2009; in Schwabe et al., 2012). According to Schwabe et al. (2012), stressful situations may modulate the processes involved in instrumental learning in a way that may produce the shift from goal-directed learning to habitual learning.

In line with these findings, it has been observed that, like cocaine cues, psychological stress induction can generate the same craving response in a cocaine abusers population (Bradley et al., 1989; Wallace, 1989; in Sinha et al., 2000). The relevance of these observations lies in the fact that both SUD and behavioral addictions (including gaming disorders, Han et al., 2011) recruit to an important extent common brain regions and produce similar physiological patterns, as quoted in the introduction of this document.

Considering the association between unhappiness and VG disorders mentioned earlier, it could be posited that the gamers concerned could not overcome the causes of their unhappiness. Indeed, studies suggest that subjects with Internet gaming disorders embark in VG play more to deal with negative affect than to achieve a good performance in the game (Schimmenti and Caretti, 2010; Billieux et al., 2013; both in Bonnaire and Baptista, 2019). In this scenario, based on the mentioned studies, a low level of happiness would imply that psychological needs are somewhat unmet and associated with the avoidant coping style together with the habit learning. Furthermore, this pattern is supported by compensatory Internet use theory, which postulates that adversity can operate as a stimulus to seek psychological comfort (i.e., satisfying the psychological needs via the cyberspace) (Kardefelt-Winther, 2014; in Dongping et al., 2016).

In other words, the psychological comfort engendered by the VG activities in this population of gamers, combined with the characteristics of the avoidant coping style (denial, social withdrawal, avoiding stressful situation, etc.) and with the traits of the habitual learning (actions’ outcomes are disregarded, with little or no awareness of actions’ consequences), might explain, at least partially, the biased perception of the emotional states in AU (*happiness* associated to VG) and of their causes of craving for VG. This assumption suggests that online gaming might not be the cause of VG addiction, but rather that VG excessive use could be a compensatory strategy to deal with pre-existing psychological characteristics and deleterious social context (Kowert et al., 2015). For instance, some studies suggest that traumatic experiences, poor emotions regulation, elements of impulsivity and the motivation to experience immersion in a virtual world would increase the likelihood of IGD and Internet addiction (Billieux et al., 2011; Schimmenti et al., 2017).

In sum, it would seem as if for AU the mentioned behavioral pattern is a manner to mitigate the difficulties to deal with stressors. This interpretation would be in line with the motives for play in problematic gaming (Demetrovics et al., 2011). Through a massive survey these authors observed seven dimensions that would cover the entire spectrum of motives for VG play in all sort of on line games: escape (from reality), cope (with stressors, playing as a way to improve mood), fantasy (trying new identities/things in a virtual world), skills development (improving concentration, coordination, new skills) recreation (relaxing aspects of gaming), competing (sense of achievement), and social (knowing/being/playing with others). This study suggests that there would be positive and beneficial motives for playing (entertaining gaming) as well as harmful ones (problematic gaming). The correlations between these factors appear to shed light on the positive and negative aspects of gaming. Whilst the weakest correlation is between escape and recreation (also low correlation was found between escape and both, skills development and competition), the strongest correlations were observed between escape and cope and fantasy. These results would indicate that *escape* and *coping* are motives associated with problematic gaming, however, the authors argue that escapism would facilitate the coping efforts to deal with stressors and negative moods. Moreover, it is noteworthy underlining that escapism had the lowest mean score in this study among the seven dimensions, which would match with the prevalence level of problematic gaming mentioned previously (Griffiths et al., 2012).

Probably, regarding AU, the accuracy in perceiving emotional states, the ability to deal with stressors and the quality of insights into oneself are dimensions that deserve much attention in the therapeutic processes.

### Therapeutic Implications

A cognitive-behavioral approach may contribute to the recovery process by enabling problematic gamers to explore the motives that lead them to abuse of VG play (Orzack et al., 2006; in Griffiths, 2008). Developing strategies to tackle stressors appears to be a therapeutic priority for treating this disorder. Consequently, this axis of work includes the understanding of the environmental demands that are perceived as exceeding the individual abilities to handle them. In this line, ensuring the accuracy of the individual’s insights into the emotional states linked to the sources of stress as well as to the game habit could increase the awareness of the underlying issues to be addressed. In particular, deciphering the conditioned desires (unconscious wanting) and the hedonic dimension (unconscious liking) (Kringelbach and Berridge, 2009; Berridge and Kringelbach, 2013) linked to VG play may produce added value information for understanding and overcoming the problematic gaming pattern. Within this frame, it could be hypothesized that distinguishing between *happiness* and *feeling alleviated* could be beneficial to the therapeutic process, although it remains to be demonstrated.

Overall, this sort of therapeutic approach may contribute to reduce the alexithymia, usually associated with this kind of disorders (Kandri et al., 2014).

In problematic internet/gaming several studies have explored and highlighted to role of alexithymia and its links with other therapeutic issues. For instance, it has been shown that alexithymic individuals are more associated with Internet addiction than non-alexthymic ones (Baysan-Arslan et al., 2016). In this research, the authors consider that the difficulty in identifying and differentiating emotions that characterizes alexithymia may lead individuals with this affliction to regulate their emotional states via their addictive activities.

Another study showed that IGD would be related with alexithymia, anxiety and depression (Bonnaire and Baptista, 2019).

Schimmenti et al. (2017) observed that traumatic experiences (mainly in males) and traits of alexithymia (mainly in females) were associated with Internet addiction symptoms, which may enable a tailored prevention and treatment approach. Besides, Internet addiction (including online role-playing) would be correlated with alexithymia, dissociation (protecting one-self in a more pleasant created reality as a means to deal with traumatic experiences) and insecure attachment (Craparo, 2011).

However, the causal link in the association between alexithymia and Internet addiction would still need to be verified, as indicated by Mahapatra and Sharma (2018). Moreover, discerning the nature of alexithymia remains an uneasy task: this emotional identification and differentiation disorder might be regarded as a stable personality trait that could increase risks of mental disorder development, and also may be seen as a defense mechanism to cope with psychological stressors (Mikolajczak and Luminet, 2006; in Mahapatra and Sharma, 2018).

Apart from alexithymia and traumatic memories, high urgency (a dimension of impulsivity defined by the proneness to have strong reactions usually tied with negative affect) and being motivated to experience immersion in a virtual world would be psychological predictors of problematic multiplayer online games (Billieux et al., 2011). These findings led the authors to posit that individuals with the two mentioned traits are more likely to use the immersion in the virtual world as a means to avoiding facing real life adverse issues. According to the authors, this behavior will lead to a deleterious outcome (culpability and embarrassment as a result of feeling unable to deal with problems), which in turn is experienced as a pernicious condition likely to activate behaviors related to high urgency and immersion.

Like the previously mentioned clinical issues, this vicious loop reinforcing escapism also appears to be a therapeutic target.

Considering the possible association between alexithymia and problematic gaming as a manner to regulate emotions (Baysan-Arslan et al., 2016; Bonnaire and Baptista, 2019), the Emotion Regulation Therapy (ERT) might strengthen the therapeutic process. The aim being that the observed difficulties in Internet (including VG) addicts to identifying emotions and regulating affects (Caretti et al., 2010; in Craparo, 2011) could be, at least partially, overcome through the ERT process. In effect, Compare et al. (2014), show that ERT operates as a means to reappraise emotions that trigger actions leading to negative consequences. Reappraising emotions is associated with the involvement of the medial prefrontal cortex, which attenuates the amygdala activation and, thus, reduces the intensity of negative affect; these two areas being coordinated by the orbitofrontal cortex (Compare et al., 2014). Since AU would be prone to associate happiness with VG play, ERT might facilitate the perceptional change enabling to link VG play with pleasure [Caretti and Craparo, 2009; in Craparo (2011) consider Internet addiction (including VG) “as a syndromic condition characterized by a recurrent and reiterated search for *pleasure* derived from dependence behavior, associated with abuse, *craving*, clinically significant stress, and compulsive dependence actions despite the possible negative consequences”]. Within this approach, it may be postulated that enabling problematic gamers to familiarize with and to see the self-transcendent notion of *happiness* could favor the distinction between pleasure and happiness and would render them less vulnerable from impulses and from environmental circumstances (Dambrun et al., 2012). The idea is to facilitate the shift from wanting more than liking (or even without liking) toward liking with little or without wanting (Berridge and Kringelbach, 2011). Furthermore, regarding motives for playing, it could be posited that helping problematic gamers to identify and distinguish the emotions tied to escaping/coping from those related to recreational gaming (Demetrovics et al., 2011), would be a necessary condition to orient effectively the ERT toward the escaping issues and targeted emotional states requiring therapeutic input. In this line, based on the previously mentioned studies in this section, it might be useful exploring the possible link that the excessive time spent in cyber activity could have with past traumatic experiences, insecure attachment, impulsivity, anxiety and depression.

In conclusion, this study suggests that the mentioned confusion of emotional states (pleasure and happiness) associated with addiction (Lustig, 2017), could take place in subjects with VG addiction, and potentially in the entire spectrum of addictions. Moreover, from a cognitive therapeutic perspective, it shows the potential benefits of reappraising emotions as a means to contribute to the emotional distortion reduction.

### Limitations

The small sample of this study demands cautiousness when making generalizations from its results. Besides, watching VG clips rather than actually playing VG might be less stimulating for chronic gamers and could have influenced the physiological values recorded during the clip visioning phases. That said, many gamers do attend to public competitions to watch other gamers playing VG. Although, to the best of our knowledge, there is no information available to affirm that there are VG addicts in these audiences.

We also faced the usual paradox when assessing craving via self-report tools. Indeed, participants were asked to judge their craving intensity for VG play whereas sensing craving often may imply a compromised self-awareness level and thus a self-assessment whose value needs to be interpreted carefully.

Although the GAS is a validated tool, which has shown its usefulness in screening addict gamers, having complemented this measurement with thorough diagnostic-driven interviews run by specialists when choosing participants to form the AU and the NAU groups would have strengthened the selection process.

The participants’ selection was centered on the gamer status (gaming addiction/non-addiction and names of games usually played) rather than on the cultural and/or educational background of the persons. Future researches could complete this approach by assessing the possible cultural and educational bias in perceiving the studied emotional states.

Moreover, including more physiological parameters related to *pleasure* and *happiness* could further complete the self-reported information and may enable reaching more robust results.

### Prospective Research

Further research is required to better understand the relationship between the studied emotional states and this addiction. For instance, since VG addiction decreases with age (Wittek et al., 2016) a longitudinal study could reveal the factors (psychophysiological, environmental, etc.) that operate that change. Moreover, VG addiction is only one area of the spectrum of addictions. Undertaking similar researches on other addictions and with larger samples could also contribute to deepening the comprehension of this issue. Finally, keep enhancing the scales that measure *pleasure* and *happiness* may provide with more accurate information about the range of nuances intrinsic to these two emotional states.

## Data Availability Statement

All datasets generated for this study are included in the article/supplementary material.

## Ethics Statement

The studies involving human participants were reviewed and approved by the Université Libre de Bruxelles Ethical Committee. The patients/participants provided their written informed consent to participate in this study.

## Author Contributions

LG developed the proposal and the conception of the original project research, searched and articulated the theoretical background, participated in the study and protocol design, elaborated the results interpretation, assembled all the chapters of the study, and in charge of the manuscript writing. ND was involved in the scientific and publication management, participated – as the Research Center Manager – in the study and protocol design, and in charge of the configuration and writing of the physiological measures. JL, as a member of the Research Center, was involved in the study and protocol design, also involved in the configuration of physiological measures, managed the experimental phases in the laboratory, and elaborated the data analysis. CL, as a full Professor at the Faculty of Psychology and Director of the Research Center for Work and Consumer Psychology, assured the scientific and publication management, participated in the study and protocol design, in charge of making the critical reviews of the manuscript along the process, and involved in the manuscript writing.

## Conflict of Interest

The authors declare that the research was conducted in the absence of any commercial or financial relationships that could be construed as a potential conflict of interest.

## Annex

### Self-Report Questionnaires

**– Six items Questionnaire: Pleasure and/or Happiness associated with VG play (Items 7 and 8 were suppressed after the preliminary phase)**

Items

(1) I enjoy playing video games.
(2) I am happy when I play video games.
(3) I would find pleasure in my video game activities.
(4) I find video games amusing.
(5) I enjoy playing my favorite video game.
(6) I often experience joy and exaltation when playing video games.
(7) I would feel pleasure when I receive praise from other people on my capacity to play video games.
(8) I don’t have fun when playing video games with other people.

fully disagree disagree slightly disagree slightly agree agree fully agree

<———I——————I——————I————————I——————I—————I———>

**– Questionnaire on Craving for playing VG**

– After having watched this clip I feel craving for playing video games.

fully disagree disagree slightly disagree slightly agree agree fully agree

<———I——————I——————I————————I——————I—————I———>

**– Three bipolar items Questionnaire: Pleasure and/or Happiness associated with VG play**

Bipolar items.

(1) I enjoy playing video games I am happy when I play video games

I——————I——————I——————I—————I

(2) I would find pleasure in I find video games amusing my video game activities

I——————I——————I——————I—————I

(3) I enjoy playing my favorite I often experience joy and exaltation video game when playing video games

I——————I——————I——————I—————I

**– Ten key words [resulting from the semantic mapping of pleasure (P) and happiness (H)]: 3/10 words to be associated with VG play**

– Joy
– Craving
– Well-being
– Impulsivity
– Fellowship
– Desire
– Fun
– Contentment
– Gratification
– Serenity

**Pleasure cluster:** joy, craving, impulsivity, desire, fun, gratification.

**Happiness cluster:** well-being, fellowship, contentment, serenity.

**– One bipolar item Questionnaire: Pleasure or Happiness associated with VG play (with explicit definitions)**

Happiness: emotional state of lasting contentment.

Pleasure: transient emotional state when satisfying a desire, a craving.

A bipolar item

## Abbreviations

AU: addict users
EEG: Electroencephalogram
ETR: Emotions Regulation Therapy
GAS: Gaming Addiction Scale
H: happiness
HR: heart rate
I.G.D.: Internet Gaming Disorders
NAU: non-addict users
OHQ: Oxford Happiness Questionnaire
P: pleasure
SHAPS: Snaith-Hamilton Pleasure Scale
VG: video games.
